# Perceptions of Physicians Regarding Implementation of Hospital Management Information Systems in a Tertiary Setting Hospital of a Developing Country

**DOI:** 10.7759/cureus.18674

**Published:** 2021-10-11

**Authors:** Rafia Hussain, Waqar Ali, Memuna Sohaib

**Affiliations:** 1 Community Medicine, Lahore Medical & Dental College, Lahore, PAK; 2 Internal Medicine, Central Park Medical College and Teaching Hospital, Lahore, PAK; 3 Dentistry, Ejaz Hospital, Gujrat, PAK

**Keywords:** hmis, advantages, disadvantages, perception, physicians, tertiary, developing

## Abstract

Background

Health Management Information Systems (HMIS) are being adopted increasingly in tertiary hospitals in developing countries like Pakistan, with the aim of improving physician and patient convenience. However, the perception of healthcare professionals regarding HMIS should be thoroughly assessed in order to achieve effective utilization and implementation of this system.

Objective

To assess the perception of physicians regarding the newly implemented HMIS in a tertiary setting hospital in Pakistan.

Materials and methods

A cross-sectional study was conducted in all the departments of Ghurki Trust Teaching Hospital (GTTH), Lahore. Total 145 physicians of varying specialty units, fulfilling the inclusion and exclusion criteria, were included and data were collected over a span of six months between July 2020 and December 2020. Data was collected by using a structured questionnaire. The SPSS software version 22 was used for data analysis. Results are presented in the form of frequencies and tables.

Results

The sample population consisted of 80 males (55.2%) and 65 females (44.8%), with the majority of the respondents having 1-5 years of clinical experience. Of the total, 61.4% (n = 89) knew about the term HMIS, while 71.7% (n = 104) of participants responded that no formal training was provided to them before the implementation of HMIS. Overall, 77.9% of respondents agreed that formal training should have been conducted before the implementation of this system. In total, 77.2% of respondents considered the ease to review patients’ history (ERPH) as the biggest advantage of HMIS, while 54.5% considered being paper-free (PF), and 53.1% considered facilitation of data analysis (FDA) as the next two major advantages. As for the disadvantages, 75.9% of respondents considered HMIS to be more time-consuming (TC) and 57.2% considered it to be a hindrance to smooth running (HSR) of clinical work in case of overloading of patients.

Conclusions

While implementation of HMIS was found to have significant advantages in the opinion of physicians, such as ease in reviewing patients' medical records, data analysis, and providing paperless patient care, it should nevertheless be preceded by formal training of all physicians regarding its use. Many physicians consider HMIS to be more TC, especially in circumstances where the patient load is overwhelming.

## Introduction

Health Management Information Systems (HMIS) is defined as "an information system especially designed to assist in the management and planning of health programs, as opposed to delivery of care" [1]. HMIS, however, is a term used to describe a comprehensive, integrated information system designed to manage all aspects of a hospital's operation, including medical, administrative, financial, and legal issues and the corresponding processing of services [2,3]. The various information systems used in hospitals can be classified as integrated or modular systems. Integrated information systems are divided into hospital general information systems, clinical information systems, management information systems, and database management systems. In another classification, HMIS is seen to include resource utilization and programming, financial management, materials and facility management, and staff management systems. HMIS is intended to improve the quality of healthcare by improving patient satisfaction, strengthening internal communication networks, reducing hospital costs, and reliably storing data in a digital environment. Above all, HMIS assures that data collection is accurate, complete, independent, and hassle-free [4].

The trend of switching from paper-based information systems to HMIS is steadily catching up in resource-limited and developing countries, such as Pakistan. Currently, HMIS is being used in Pakistan in both public sector and private sector hospitals. In Pakistan, this was adopted in the early 1990s by the HMIS cell in the Ministry of Health. This system has now been implemented in a phase-wise manner and more than 90% of primary healthcare facilities are currently using it [5]. However, the trend is somewhat lacking in tertiary healthcare facilities and only a few hospitals have managed to convert to this system [6]. Ghurki Trust Teaching Hospital (GTTH), a 600-bedded tertiary setting hospital in Lahore, Pakistan, has recently converted operations to a state-of-the-art HMIS.

As evident from the recent literature and upcoming trends, HMIS explicitly is the utmost necessity of hospital data generation and analysis. On the narrower perspective, it provides surmountable cost-effectiveness to the particular hospital setting, allowing efficient intra-institutional correspondence by the flow of correct information through the chain of command, better communication between the stakeholders, timely supply of materials, and in-house and inter-institutional information transfers to healthcare staff, resulting in improved efficacy of the hospital both in terms of time and cost [2]. From a broader perspective, it boosts the efficacy of the healthcare system as a whole. It opens fine opportunities for the generation of baseline level statistics both at grass root and national levels, opening new arenas for health and healthcare researches and eventually contributes to evidence-based medicine practice [7].

At every level, starting from designing to implementation, monitoring to quality assurance, various challenges and concerns arise that lead to a weak HMIS implementation. Some of these are weak planning and financing, misuse of resources, weak monitoring and supervision, poor skills development, poor quality data, and lack of leadership and commitment [2,8]. During the implementation phase, the healthcare workers form the main "actors", whose commitment and understanding about the new system is crucial to the successful implementation. The key potential barriers perceived by the healthcare workers while dealing with the HMIS include high initial cost and uncertain financial benefits, high initial physician time costs due to tedious and cumbersome technology, difficult complementary changes and inadequate support and electronic data exchange, lack of incentives, and physicians’ attitudes itself. The perceived potential benefits however regarding the use of HMIS for clinicians are easy and efficient viewing of records and data, effective documentation and care management, timely ordering of labs and services, messaging along the chain of command, accurate analysis and reporting system, patient-directed functionality and transparent billing [9].

In the light of the above, all the constraints in the effective utilization and efficient implementation of HMIS should be minimized. Healthcare workers form the core of the healthcare system, hence any novel system/technology being introduced in the healthcare system should target their convenience. Moreover, knowledge regarding its utility, and perception regarding its short and long-term effectiveness should be clearly communicated to them. The hospital management and other stakeholders should be well abreast with all the problems faced by the healthcare workers in dealing with this newly implemented system of data generation and analysis so that timely intervention can be done to address the issues. This study aims to highlight the perception of doctors regarding the implementation of HMIS in their setting, encompassing the problems they face while dealing with the system. It is expected to provide useful suggestions and recommendations to the management authorities in order to address the core issues regarding the effective implementation of the system.

## Materials and methods

A cross-sectional study was conducted in all the departments of GTTH. GTTH is a 600-bedded hospital, located in the peri-urban area of Lahore. It is an ISO 9001 - 2015 certified hospital, affiliated with Lahore Medical & Dental College (LMDC), recognized by the Pakistan Medical Commission (PMC), and affiliated with the University of Health Sciences (UHS). The various departments included in the study were Medicine, Dermatology, Gynecology and Obstetrics, Surgery, Urology, Otorhinolaryngology (ENT), Emergency Medicine, Radiology, Pediatric Medicine, Anesthesia, Ophthalmology, and Orthopedics. The total duration of the study was six months, from 1st July 2020 to 31st December 2020. For this purpose, due approval was acquired from the IRB of LMDC and GTTH (Approval number LM&DC 12066-67/2020).

Data was collected using a self-administered structured questionnaire, which was designed in English as well as the Urdu language. The questionnaire comprised close-ended questions divided into three sections: the first section covering the basic demographic profile/biodata of the participating physicians; the second consisting of questions related to basic knowledge regarding HMIS and also the questions regarding formal training, usage, and perception regarding the IT and supporting staff, and the third covering the perception of physicians regarding advantages and disadvantages of the HMIS.

Physicians of all designations working in the hospital, including house officers, medical officers, postgraduate residents, and consultant physicians were included in the study population, which consisted of 248 physicians. Non-probability convenience sampling technique was used to determine the sample population. The sample population for the study comprised 145 physicians, working in the aforementioned departments of GTTH. The informed written consent for participation in the study was obtained from each physician. Only those physicians who declined giving consent for participation and those who did not have official access to HMIS were excluded from the study. Confidentiality of biodata was ensured.

The data after cleansing was entered in and analyzed using the SPSS Version 22. Biodata of physicians and their perception regarding a particular question was presented in the form of frequencies and percentages. The Chi-square test was applied to observe the effect of designation, gender, years of clinical experience (categorical), and the previous experience of HMIS outside GTTH.

## Results

Data were collected from a total of 145 clinicians, including 80 (55.2%) males and 65 (44.8%) females. Total 104 (71.7%) clinicians had a clinical experience of 1-5 years, whereas 18 (12.4%) had less than one year of clinical experience. The number and percentage of clinicians with respect to the individual specialty are shown in Table 1.

**Table 1:** Department or specialty-wise distribution of study participants.

Departments		Frequency	Percent
	Dermatology	3	2.1
	Gynecology and obstetrics	25	17.2
	Internal medicine	22	15.2
	Surgery	22	15.2
	Urology	4	2.8
	ENT	11	7.6
	Emergency medicine	4	2.8
	Radiology	9	6.2
	Pediatric medicine	6	4.1
	Anesthesia	9	6.2
	Ophthalmology	4	2.8
	Orthopedics	26	17.9
	Total	145	100.0

The participants included 41 (28.3%) house physicians/ internees, 25 (17.2%) medical officers, 59 (40.7%) postgraduate resident trainees, and 20 (13.8%) consultant physicians. Of the total, only 23 (15.9%) doctors had had previous experience in the use of HMIS. Total 89 (61.4%) doctors responded correctly when asked to amplify the term "HMIS" to its full form. Among the total, 67.6% of clinicians believed that the current version of HMIS was satisfactory and required no changes.

The majority of the respondents (60%) did not consider the system to be cost-effective compared to the traditional paper-based system, and a similar number (63.4%) disagreed that this led to a decrease in the number of physician-based errors at all. The major advantages of HMIS, as per the opinion of the respondents, were it being paper-free (54.5%), making the review of patient history simpler (77.2%), and facilitation of data analysis (53.1%), while the major disadvantages reported included it being time-consuming (75.9%) and causing hindrance in patient care in times of patient overload (57.2%). The major advantages and disadvantages of HMIS in the opinion of the respondents are shown in detail below (Tables 2-3).

**Table 2:** Perception of physicians regarding the advantages and disadvantages of HMIS shown as the frequency and percentage of the responses on the study questionnaire. HMIS: Health Management Information Systems

Serial number	In your opinion the advantages of HMIS are?	Answers	
		Yes	No
1.	Cost-effectiveness	58 (40%)	87 (60%)
2.	Being paper-free	79 (54.5%)	66 (45.5%)
3.	Patients do not have to save prescriptions	75 (51.7%)	70 (48.3%)
4.	Easy to review patients’ history	112 (77.2%)	33 (22.8%)
5.	Fewer chances of error on part of clinicians and mixed reports	53 (36.6%)	92 (63.4%)
6.	Helps in identifying the fault or negligence of health professionals	54 (37.2%)	91 (62.8%)
7.	Helps in the generation and sharing of data	52 (35.9%)	93 (64.1%)
8.	Helps in reviewing the attendance/leaves of the health professionals	63 (43.4%)	82 (56.6%)
9.	Facilitates data analysis	77 (53.1%)	68 (46.9%)

**Table 3:** Perception of physicians regarding the disadvantages of HMIS shown as frequency and percentage of the responses on the study questionnaire. HMIS: Health Management Information Systems

Serial number	In your opinion the disadvantages of HMIS are?	Answers	
		Yes	No
1.	It is time-consuming	110 (75.9 %)	35 (24.1%)
2.	It causes hindrance to smooth running in case of overloading of patients	83 (57.2%)	62 (42.8%)
3.	Healthcare professionals feel threatened due to the fear of being identified in case of error	29 (20%)	116( 80%)
4.	Poor doctor-patient contact and confidence building	66 (45.5%)	79 (54.5%)
5.	Require computer skill	69 (47.6%)	76 (52.4%)
6.	Data can be misused	32 (22.1%)	113 (77.9%)
7.	Data loss due to technical and computer errors	47 (32.4%)	98 (67.6%)

The majority (71.7%, n = 104) of the doctors responded that no formal training was imparted prior to implementation of HMIS, and 77.9% were of the opinion that such training was essential for effective implementation of the system. Of note, 91% of respondents did not know how to raise and report an "incident" through HMIS. Total 94 (64.8%) respondents believed that the system was being maintained properly and 116 (80%) were of the opinion that the staff of the hospital information technology (IT) department designated to assist in case of any technical problems, was efficient and co-operative.

The Chi-square test revealed a significant association of prior training of HMIS use with the belief that the current system was being maintained properly. Out of the total 104 (71.7%) physicians who had not received prior training in HMIS, 58.6% (n = 61) believed that the system was being maintained properly, while 80.5% (n = 33) of those with prior training believed that the system was properly maintained (p-value 0.013). Out of the total physicians who thought that the system was being maintained properly, 95.4% believed that the IT department staff was efficient and cooperative (p-value 0.001), thus there was a strong association between the two. The Chi-square test revealed that clinicians with prior experience with the use of this system associated it with more advantages (p-value 0.03), while also believing that the system was well maintained (p-value 0.031). Moreover, the knowledge about incident raising had a strong association with the gender of the respondent (p=0.025). The designation of doctors also had a strong association with knowledge about the name of HMIS (p = 0.025) and a very strong association (p=0.000) with the provision of formal training about HMIS.

## Discussion

Electronic HMIS is being adopted in healthcare setups all over the world with the aim of improving administrative efficiency. In developing countries such as Pakistan, HMIS is being inculcated in such setups gradually, while traditional paper-based system predominates in both public and private sectors. This implementation phase requires a significant change of practices and mindsets and thus brings with it certain challenges and hiccups, which are important to address. It calls for an immense commitment on part of all the stakeholders; their acceptance of the challenges that the system can bring, and more importantly, their willingness to confront and resolve these challenges. Physicians who are at the forefront of patient encounters should be able to utilize this system as a tool for collaborative patient care, data synthesis and analysis, and a tool to improve the efficiency of the hospital both in terms of cost and care [8]. This study discovers that physicians working at a tertiary setup, where the system has recently been implemented, have a myriad of concerns related to this system.

In our study, there was unprecedented unanimity of thought in physicians with regard to the need for formal training prior to implementation of HMIS for full utilization of its potentials. A majority of our respondents denied being given formal training prior to implementation of HMIS in the hospital, which reflected in poor awareness of certain important features. For instance, when asked regarding a specific feature of "incident raising", only 9% of the respondents were able to answer correctly. This important finding of the study implies that the system was not being utilized to its full capacity, and the users were unaware of the various features, attributable to the absence of formal training sessions before, which is very similar to the previous studies [10,11]. However, the previous studies also show that the clinicians believe that work-in trainings are also very important, where the clinicians are given demonstrations if ever they face any difficulty.

Several advantages of HMIS have long been established. However, the perceptions of doctors from GTTH regarding those were variable in this regard. The majority of the physicians were of the opinion that this system is not cost-effective, neither did they believe that it has an added advantage in preventing clinicians' error with regards to patient identification and medical reports. While using HMIS, all the documentations are made chronologically with no option to change. Hence, the point of negligence in the chain of command can be accurately identified. However, in our study, only 37% of the participants believed that this system would be able to achieve this purpose.

Patient care has long been a multidisciplinary integrated activity in a tertiary care hospital where frequent discussions and referrals to other departments are made in order to reach a holistic management plan. This approach calls for an easy review of history and investigations at the convenience of the clinicians. A great number of respondents in our study believe that the review of patient history is much easier and hence supports the collaborative approach to patient care which is also consistent with the belief of clinicians from other similar studies [10,11].

Hospital data or records, though not representative of the community or population health status, are however a very significant source of information about the epidemiology of the diseases in that population. HMIS is a reliable source of accurate data generation, easy dissemination to those in need, and facilitates convenient data extraction in case of analysis either by the hospital itself or by the government bodies. However, the majority of our respondents thought contrary to this viewpoint. Half of the sample believed that being paper-free is an advantage of HMIS.

The benefits of HMIS can extend to hospital staff and physicians as well, such as ease of submitting daily attendance and leave requests [10]. However, more than half of our participants did not perceive this as advantageous.

Literature has also shown the existence of numerous disadvantages of HMIS. A very common one according to our respondents was it being more time-consuming. We believe that this may be attributable to the skill and speed required with keyboard and screen use, which is dependent on personal experience and expertise. It may thus be more time-consuming for individuals with less adaptation to this modality and hence result in choked clinics, outpatient departments, and slowing down of day-to-day ward routine due to sluggish documentation and data entry. This would directly result in long patient waiting time, reduced doctor-patient interaction, and henceforth patient dissatisfaction. Though this is understandably due to lack of computer skills and training, yet more than half of our respondents are of the opinion that HMIS does not require any extraordinary computer skill proficiency neither do they believe that there is any disadvantage in case of loss of data due to technical errors.

Clinical setting ethics and medical practice demand "active listening" and "exclusivity" by the doctors, which in the opinion of the authors is compromised by the computer screen walling off the clinician from the patient. The physician may seem more preoccupied with typing and documentation, thus reducing direct eye contact with the patient which may, in turn, lead to patient dissatisfaction. While this may seem like a major disadvantage, less than half of our participants viewed this to be actually true.

Misuse of the data is also a very debatable point in the case of the HMIS data entry system. A large majority of our participants did not think that the data can be misused while using HMIS. The limited and controlled access to the data is probably the solution to this problem. Similar to the previous studies, our respondents also believe that new tools should be added to the HMIS in order to improve it [10,11].

Hospital job for clinicians is more of a mobile activity where the time is spent moving back and forth between patient bedside, nursing counters, duty rooms, and other departments for patient evaluation. Hence, the desktop at the nursing counter or one single computer per ward is not sufficient enough for the doctor. Hence, one of our respondents was of the view that HMIS should be given access to android mobile phones as well, similar to the previous studies [10].

HMIS is a computer-based system, hence the collaborative efforts of the IT department remain a cornerstone in its successful implementation. Similar to the previous studies, the vast majority of respondents believe that the IT department at GTTH and their staff are very efficient and cooperative in case of any technical error or difficulty, responding immediately to a phone call or complaint [10].

In medical science, we all know that nothing is absolute with new diseases emerging every other day, novel investigations being devised, new drugs added to the pharmacy, and new management techniques being devised. Moreover, every case is a new case hence, the system demands to be maintained/upgraded [10]. The majority of the clinicians believe that the system in GTTH is being properly maintained and upgraded as per se the clinicians' or users' requirements.

The study sample had representatives from all the departments, including both the junior and senior doctors, and the study was novel in this clinical setting. However, certain limitations need to be acknowledged. Owing to financial constraints, the study did not include the public hospitals and primary and secondary healthcare outlets, where the HMIS is also in its initial stages, with different kinds of working environments and patient loads. The sample size is not large enough, so generalizability is low. Also, the study sample does not include other healthcare workers and management and supporting staff, whose problems and concerns would significantly influence the success of this system. In the future, studies should be conducted with a larger sample size including other healthcare workers, viz nurses, phlebotomists, radiology technicians, and management officers in order to inculcate their perspective in this domain.

## Conclusions

Indeed, HMIS implementation is the way forward for efficient management of hospital services, however, as this study reveals, it has its sets of benefits as well as limitations. The study concludes that ease to review patients’ medical history is the most common perceived advantage of HMIS among physicians, followed by the provision of a paper-free system and facilitation of data analysis. On the other hand, the major perceived disadvantages of HMIS were it being more time-consuming and thus a possible hindrance to smooth running of clinical work in case of increased patient flow. Formal training and prior experience of use tend to boost physician confidence in the system, and the presence of supportive technical staff is also pivotal in this aspect. The study sample has representatives from all the departments, including both the junior and senior doctors, and the study is novel in this clinical setting. In the future, studies should be all-inclusive and include representatives from paramedical, nursing, administrative, and other nonclinical staff of the hospital.

## References

[1] World Health Organization (2004). Developing health management information systems: a practical guide for developing countries. https://iris.wpro.who.int/bitstream/handle/10665.1/5498/9290611650_eng.pdf.

[2] Balaraman P, Kosalram K (2013). E-hospital management & hospital information systems-changing trends. IJIEEB.

[3] Abdulla MN, Al-Mejibli I, Ahmed SK (2017). An investigation study of hospital management information system. IJARCCE.

[4] Demirel D (2017). Hospital management information systems in health sector and development in Turkey. J Curr Res Health Sec.

[5] Qazi MS, Ali M (2009). Pakistan's health management information system: health managers' perspectives. J Pak Med Assoc.

[6] Ujan IA, Bhutto A, Rind MM, Suhaimi MA (2016). Acceptance of HMIS by healthcare professionals of private sector hospitals of Pakistan. SURJ (Science Series).

[7] Shaikh BT, Rabbani F (2005). Health management information system: a tool to gauge patient satisfaction and quality of care. East Mediterr Health J.

[8] Berg M (2001). Implementing information systems in health care organizations: myths and challenges. Int J Med Inform.

[9] Miller RH, Sim I (2004). Physicians' use of electronic medical records: barriers and solutions. Health Aff (Millwood).

[10] Lium J-T, Tjora A, Faxvaag A (2008). No paper, but the same routines: a qualitative exploration of experiences in two Norwegian hospitals deprived of the paper based medical record. BMC Med Inform Decis Mak.

[11] Meyer TA (2000). Improving the quality of the order-writing process for inpatient orders and outpatient prescriptions. Am J Health Syst Pharm.

